# The dynamic association of body mass index and all-cause mortality in multiple cohorts and its impacts

**DOI:** 10.1186/1742-7622-11-17

**Published:** 2014-10-24

**Authors:** Jianghua He, Qing Yu, Huiquan Zhang, Jonathan D Mahnken

**Affiliations:** 1 Department of Biostatistics, University of Kansas Medical Center, Mail Stop 1026, 3901 Rainbow Blvd, 66160 Kansas City, KS, USA; 2 Sanofi Pasteur Research & Development in China, Beijing, China

**Keywords:** Cox model, Non-proportional hazards, Time-dependent covariates Cox model, Length of follow-up

## Abstract

**Background:**

In the literature, different shapes of associations have been found between body mass index (BMI) and mortality and some of the findings were opposite to each other. The association of BMI and mortality in a single cohort has been found to be dynamic that can lead to different findings under different settings. The identified dynamic features were consistent with the heterogeneity in the literature. It is meaningful to find out whether such dynamic associations exist in other populations.

**Methods:**

Data of six different cohorts were used for analysis and comparison. The proportional hazards assumptions for BMI in Cox models were tested to identify dynamic associations in each cohort. Time-dependent covariates Cox model was used to model the association of BMI and mortality risk as functions of follow-up time. The Cox model was applied to the pooled data with survival times censored at 5 to 40 years to show the potential impact of the dynamic association on traditional Meta-analysis.

**Results and discussion:**

Dynamic associations were identified in six models (4 for men and 2 for women), four of which showed the same changing pattern: the elevated mortality risk for low BMI decreased while that for high BMI increased with follow-up time. When the Cox model was applied to the pooled data excluding the largest and also the shortest cohort, low BMI was but high BMI was not associated with high mortality for men with censoring at 5 years but the association for low BMI became weaker and that for high BMI became much stronger when censoring time was at 40 years. The dynamic association indicated that shorter studies tend to obtain inverse associations between BMI and mortality while longer studies tend to obtain J-shaped associations.

**Conclusions:**

Different or even opposite results about body weight and mortality in the literature may be in part due to the underlying dynamic association of BMI and mortality. The dynamic features need to be taken into consideration in future studies.

## Background

With the prevalence of obesity exceeding epidemic proportions and continuing to escalate [1], it is urgent and challenging for researchers to identify the health impact of obesity. A popular epidemiologic approach of measuring the impact of obesity is through examining the association of body mass index (BMI) and mortality. In the literature, BMI and mortality was mostly reported to be associated as J-shaped [2-7] or U-shaped [8,9], suggesting that both high body weight and low body weight are associated with elevated mortality risk. Other types of associations, such as no association [2-4], direct associations [10-12], or even inverse associations of BMI and mortality [13-17], were also found. The heterogeneity among these findings created controversies about study designs and analysis approaches.

Some of the controversies are about the validity of using baseline BMI for analysis. A study based on the First National Health and Examination Survey (NHANES I) showed a steep decrease in BMI among the elderly toward the end of lifespan [18]. Some researchers argued that individuals may lose a large amount of weight due to preclinical diseases before they died [19-21]. So, baseline BMI may not represent real weight status, especially for elderlies or sick individuals who died soon after enrolling in a study. A proposed strategy is to use BMI at earlier days or the average of BMI at different ages rather than baseline BMI [18,22,23]. Compared with the difficulty of obtaining BMI measures prior to baseline, a much easier approach is to set back the actual study period by excluding deaths within early years of follow-up. Dropping early deaths within 3 to 6 years after baseline has become a commonly used strategy for sensitivity analysis. However, a large scale Meta-analysis did not find a significant impact of dropping early deaths in 4–6 years [24].

Some studies were conducted to explore the system dynamics of the association of BMI and mortality. Simulation studies designed based on the Framingham Heart Study (FHS) showed that the association of BMI and mortality estimated using logistic regression models for the same group of individuals may depend on when BMI is measured as well as how long these subjects are followed [25,26]. When the proportional hazards (PH) assumption was violated in the Cox model for FHS men, the association of BMI and mortality was estimated as a time-varying curve of BMI rather than a single curve of BMI (or several hazard ratios/odds ratios, one for each weight group defined by BMI range) as most studies in the literature did. The time-varying curve was decreasing in BMI at baseline (at the beginning of the study, only individuals with low BMI had relatively higher immediate mortality risk) and gradually turned J-shaped after about 40 years of follow-up (forty years later, survivors with baseline BMI at both ends had higher immediate mortality risk) [26]. The dynamic survival model provided a systematic view on the association of baseline BMI and mortality and can explain the impacts of length of follow-up and the measurement time of BMI on findings using logistic regression models.

The dynamic association of BMI and mortality found in FHS men suggested that shorter studies are more likely to obtain inverse associations of BMI and mortality when traditional methods, such as logistic regression or Cox models are used. This is consistent with the fact that all the inverse associations we found in the literature were based on cohorts with no more than 10 years of follow-up [13-17]. However, the findings about dynamic association were based on a single cohort (FHS) and may not be generalized to other populations.

This study aims to examine whether the dynamic association found in the original cohort of FHS exists in other populations. If dynamic associations are found in these populations, further analyses will be conducted to explore the impacts of such dynamic associations on results about body weight and mortality obtained with traditional epidemiological methods, such as Meta-analysis on pooled data.

## Methods

### Data

The six cohorts are the Original and Offspring cohorts of FHS (https://www.framinghamheartstudy.org/), the Charleston Heart Study (CHS, http://www.icpsr.umich.edu/icpsrweb/ICPSR/studies/4050?geography=South+Carolina), NHANES I and II (http://www.cdc.gov/nchs/nhanes.htm), and National Health Interview Survey (NHIS, http://www.cdc.gov/nchs/nhis.htm). For the Original Cohort of FHS, 5209 subjects were included and examined every other year starting from1948. For this study, only subjects examined at Exam 4 were included for analysis and Exam 4 was considered as the baseline because the method of measuring some variables was not standardized until Exam 4. The total number of observations was 164,052 with baseline measures of covariates and time to all-cause mortality. We excluded 2,503 individuals with missing BMI or current smoking status. We also excluded 85 individuals with BMI less than 14 or greater than 60. The final dataset has a total of 161,464 Black, White, or Hispanic individuals with mean follow-up of 9.0 years and standard deviation of 5.6 years (range: 0.003 - 38.2 years).

### Statistical analysis

#### Dynamic associations of BMI and mortality in individual cohorts

The analysis approach used in a previous study [26] of the dynamic association of BMI and mortality in the Original Cohort of FHS was modified for this analysis. Men and women were analyzed separately as the dynamic association of BMI and mortality was found to be stronger in men than in women [25,26].

The dynamic association was examined at first by testing the proportional hazards (PH) assumptions of BMI when the Cox model was used to model time to death within each cohort. Because BMI is highly right skewed, Lean BMI (LBMI = 1/BMI) was used for modeling the curvature association of BMI and mortality [26]. The dynamic association of BMI and mortality was determined by testing the PH assumptions of both the linear and quadratic terms of LBMI based on the scaled Schoenfeld residuals [27]. If the PH assumption for any of the two LBMI terms was violated at 0.05 level (p < 0.05), the dynamic association of BMI and mortality was considered significant. If the PH assumption for any of the two LBMI terms is violated at the 0.1 level, the dynamic association of BMI and mortality was considered a borderline case.

Time-dependent covariate Cox models were used to model significant or borderline dynamic associations identified in the previous step. Just as most epidemiological studies, no repeated measures of any covariate was used besides baseline characteristics. The time-dependent covariate Cox model was used as an approach of modeling the time-varying coefficient of a covariate by adding an interaction between the covariate and follow-up time. When the PH assumption of LBMI was violated, the linear interaction of LBMI with follow-up time was estimated; when the PH assumption of the quadratic term of LBMI was violated, the linear interactions of both LBMI terms with follow-up time were estimated. For visual comparison, the hazard functions for BMI at different time points of follow-up were plotted together to show the changing pattern of the association with time. All hazard functions of BMI were scaled for a better visual comparison (see Additional file 1 for more details).

#### Meta-analysis using cox model and changing length of follow-up

The impact of dynamic associations on traditional analyses was explored by examining whether the result of Meta-analysis on pooled data changes when the length of follow-up changes. For traditional Meta-analyses with individual data available, subjects from different cohorts were pooled together as a comprehensive database even though some of the cohorts have very different lengths of follow-up. With all cohorts combined, the Cox model was applied when the survival times of all subjects in the combined data were right-censored at 5, 10, 15, 20, …, 40 years, respectively. Nobody had survival time greater than 40 years in any of the six cohorts, so that the last model represents the result of a traditional Meta-analysis. The estimated BMI hazard ratios were compared across all circumstances to show the potential impact of length of follow-up.

Besides BMI related terms and stratification by sex, major confounders such as age and smoking status (current smoker vs. non-smoker), and race (Black, Hispanic vs. White) at baseline were included in all models (details of the model is in Additional file 1). These covariates were chosen because they are well known confounders for mortality and were considered in most studies in the literature. We did not include covariates such as blood pressure, cholesterol, and triglycerides as they could be affected by body weight during the pathway of affecting mortality [28]. Comorbidities such as heart diseases, cancer, or diabetes at baseline were not included in these studies either as these diseases could also be the consequences of body weight’s impact on health status [29-31]. Covariates such as life styles and social economic status were not available in the database used for our study. For Meta-analysis, the differences among different cohorts were also considered by adding indicator of study cohorts. All subjects with time to death or end of follow-up, survival status at the end of follow-up, gender, age, race, and smoking status available were included for analysis.

For sensitivity analysis, the same set of analyses within cohort was repeated with subjects died within 4 years of follow-up excluded; Meta-analysis excluding NHIS cohort was conducted to examine the sensitivity of the analysis to a cohort with the dominant sample size yet the shortest follow-up. All analyses were conducted with STATA 11.1 (StataCorp, College Station, Texas, USA).

## Results

The six cohorts included in this study are all well-known and together can represent the broad range of cohorts used in the literature. Table 1 shows the summary statistics of the six cohorts. Charleston Heart Study was the smallest cohort (n = 1,952, 1.21% of all cohorts combined) yet with the second longest follow-up (median = 26.3 years). NHIS had the shortest length of follow-up (median = 7.1 years) but its sample size (n = 125,159) was the largest among all cohorts (79.5% of all cohorts combined). The large variation among cohorts in terms of age composition, mortality rate, smoking status, race, and BMI were noticeable. For example, both cohorts of FHS had white participants only, Charleston, NHANES I and II had white and black participants, while NHIS had black, white, and a relatively small group of Hispanic participants (n = 3,161, 2.5% of NHIS and 2.0% of all cohorts combined) as well.

**Table 1:** Summary statistics (counts and percentages for categorical variables and median, minimum, and maximum for continuous variables) for variables under consideration in six cohorts

**Cohort**	**Charleston n = 1,952**	**Framingham original n = 4,526**	**Framingham offspring n = 4,444**	**NHANES I, n = 13,153**	**NHANES II, n = 9,069**	**NHIS n = 128,320**	**Total n = 161,464**
**Variables**							
Male	850 (43.6%)	2,005 (44.3%)	2,174 (48.9%)	5,327 (40.5%)	4,254 (46.9%)	53,910 (42.0%)	68,520 (42.44%)
Black	741 (38.0%)	0 (0%)	0 (0%)	1910 (14.5%)	994 (10.96%)	17,434* (13.93%)	21,079 (13.05%)
Smoker	989 (50.7%)	2,352 (52.0%)	1,988 (44.7%)	4,294 (32.7%)	2,940 (32.4%)	35,607 (27.8%)	3,161 (1.96%)
Death	1,129 (57.8%)	2,764 (61.1%)	444 (10.0%)	4,237 (32.2%)	2,110 (23.3%)	11,568 (9.1%)	22, 252 (13.78%)
Age (years)	48 (35, 97)	49 (34,69)	38 (25, 62)	48 (25,75)	58 (30,75)	45 (25, 90)	46 (25, 97)
BMI (kg/m^2)	24.6 (14.8, 52.9)	25.5 (15.9, 56.7)	25.1 (16.5, 54.9)	25.0 (14.0, 58.7)	25.4 (14.1, 59.9)	24.4 (14.0, 59.4)	24.7 (14.0, 60.0)
Survival time** (years)	26.3 (0.03, 30.7)	30.1 (0.09, 38.2)	19 .4 (0.01, 22.9)	18.3 (0.03, 22.1)	14.3 (0.02, 16.8)	7.1 (0.003, 9.0)	7.5 (0.003, 38.2)

*Besides black and white participants, NHIS had 3,161 Hispanic participants.

**Survival time: time to death (or end of follow-up) from baseline.

### Cox models and proportional hazards assumptions for individual cohorts

**Figure 1:**
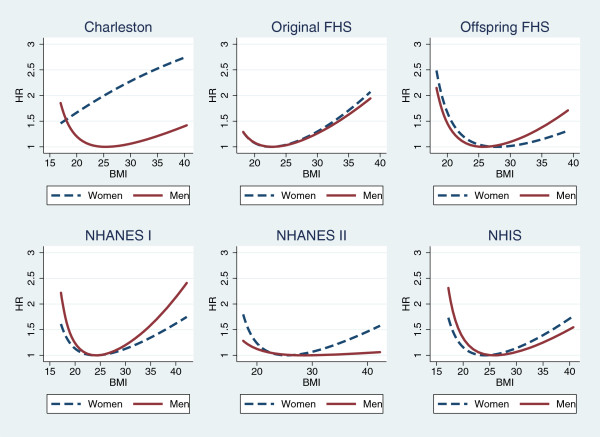
**Hazard ratios of BMI based on Cox models for individual cohorts.** For each curve, the hazard ratio at the vertex is 1 (control point). The vertex for Charleston Women is outside of the data range so that the curve looks monotonic. Each line is plotted within the range of 1st - 99th percentiles of BMI at baseline.

When the Cox model was used, most models identified a J-shaped or U-shaped association for BMI and mortality. Table 2 shows the estimated log hazard ratios and the p-values of the tests of significance of all variables. Most models showed quadratic associations of LBMI and mortality except that the model for Charleston women showed a direct association while that for NHANES II men showed no association of LBMI and mortality. Figure 1 presents the hazard ratios of BMI for the models in Table 2. Most models support U- or J-shaped association except that the association is direct for Charleston women (higher the BMI, higher the risk) and that for NHANES II men is almost flat (mortality risk is not associated with BMI).

**Table 2:** **Estimated cox models**^**+ **^**(estimated means and standard errors of log hazards ratios, p-values of testing covariate effects, and p-values of testing the proportional hazards assumptions) by cohort and gender**

	**Charleston**		**Framingham original**		**Framingham offspring**		**NHANES I**		**NHANES II**		**NHIS**	
	**Female 580/1,102**	**Male 549/850**	**Female 1,371/2,521**	**Male 1,393/2,005**	**Female 145/2,270**	**Male 299/2,174**	**Female 1,966/7,826**	**Male 2,271/5,327**	**Female 874/4,815**	**M 1,236/4,254**	**Female 6,043/7,4410**	**M 5,525/5,3910**
Age (5 years)	0.47 (0.02)	0.40 (0.02)	0.52 (0.02)	0.48 (0.02)	0.52 (0.05)	0.55 (0.03)	0.48 (0.01)	0.46 (0.01)	0.49 (0.02)	0.49 (0.02)	0.45 (0.01)	0.42 (0.01)
	<0.001	<0.001	<0.001	<0.001	<0.001	<0.001	<0.001	<0.001	<0.001	<0.001	<0.001	<0.001
	0.48	0.60	0.01	0.40	0.81	0.53	0.04	0.004	0.46	0.03	0.02	0.001
Non-Smoker	1.00	1.00	1.00	1.00	1.00	1.00	1.00	1.00	1.00	1.00	1.00	1.00
Smoker	0.37 (0.09)	0.61 (0.10)	0.33 (0.06)	0.34 (0.06)	1.01 (0.18)	0.68 (0.12)	0.43 (0.06)	0.42 (0.05)	0.65 (0.08)	0.70 (0.07)	0.65 (0.03)	0.58 (0.03)
	<0.001	<0.001	<0.001	<0.001	<0.001	<0.001	<0.001	<0.001	<0.001	<0.001	<0.001	<0.001
	0.73	0.75	0.45	0.05	0.23	0.42	0.30	0.005	0.08	0.03	0.69	0.10
White	1.00	1.00	1.00	1.00	1.00	1.00	1.00	1.00	1.00	1.00	1.00	1.00
Black	0.41 (0.09)	0.20 (0.09)	NA	NA	NA	NA	0.32 (0.06)	0.18 (0.06)	0.09 (0.11)	−0.09 (0.09)	0.23 (0.04)	0.30 (0.04)
	<0.001	0.02					<0.001	0.002	0.42	0.31	<0.001	<0.001
	0.89	0.56					0.37	0.79	0.09	0.68	0.85	0.76
Hispanic	NA	NA	NA	NA	NA	NA	NA	NA	NA	NA	−0.20 (0.13) 0.13 0.78	−0.23 (0.12) 0.06 0.43
LBMIc*	−1.89 (0.53)	0.49 (0.69)	−2.04 (0.42)	−1.79 (0.54)	1.79 (1.21)	4.72 (11.29)	−0.45 (0.28)	−0.58 (0.33)	0.11 (0.43)	0.49 (0.51)	−0.24 (0.16)	1.00 (0.21)
	<0.001	0.48	<0.001	0.001	0.14	0.68	0.11	0.08	0.80	0.34	0.14	<0.001
	0.23	0.60	0.07	0.04	0.41	0.71	0.23	<0.001	0.58	0.85	0.61	0.001
LBMIc^2^**	1.33 (4.74)	16.31 (7.67)	21.79 (4.17)	20.71 (5.64)	25.74 (11.84)	30.46 (12.35)	17.45 (2.24)	28.28 (2.95)	18.55 (3.41)	4.94 (3.83)	19.97 (1.18)	22.19 (1.56)
	0.78	0.03	<0.001	<0.001	0.03	0.01	<0.001	<0.001	<0.001	0.20	<0.001	<0.001
	0.35	0.87	0.44	0.75	0.10	0.08	0.81	0.22	0.002	0.44	0.61	0.48

^+^These are Cox regression models with all the covariates in the table included simultaneously.

*LBMIc: LBMI centered by its mean of all individuals included in each model and multiplied by 10. The variable was scaled (multiplied by 10) so that the coefficients are not too large.

**LBMIc^2^: the square term of LBMIc.

In each cell, the value at the bottom row is the p-value of testing the proportional hazards assumption and any p-value <0.05 was considered significant and 0.05 < =p-value <0.1 was considered a borderline case (with three rows).

Table 2 also shows the results of testing the proportional hazards (PH) assumptions of those Cox models. For men, analyses in the Original Cohort of FHS, NHANES I, and NHIS yielded significant dynamic associations of BMI and mortality as the PH assumption for linear LBMI or quadratic LBMI was violated; the model based on men of the Offspring Cohort of FHS is a borderline case with p = 0.08 for the quadratic term of LBMI. For women, NHANES II has a significant dynamic association of BMI and mortality and the Original Cohort of FHS is a borderline case with p = 0.07. No dynamic association between BMI and mortality was found for Charleston Heart Study, which may be because of the small sample size.

### Dynamic associations of BMI and mortality in individual cohorts

**Figure 2:**
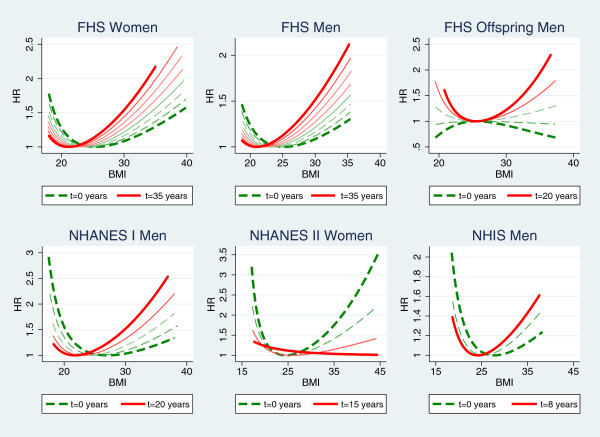
**Hazard ratios of BMI at different time points of follow-up for cohorts with significant (p < 0.05) or border case (0.05 ≤ p < 0.1) dynamic associations of BMI and mortality.** Each line was plotted within the range of 1st - 99th percentiles of BMI of all individuals remain alive at the time point. The curves for the first time point (0 years) are in dashed thick lines and those for the last time points (determined by the maximum length of follow-up of each cohort) are in solid thick lines and those of in-between time points are in thin lines. Gaps between consecutive curves (time points) are 5 years except for NHIS men (the three curves are for 0, 5, and 8 years) as the longest follow-up is 9 years in this cohort.

The hazard ratios of BMI at different follow-up time points for all six models with significant or borderline dynamic associations were plotted for comparison in Figure 2. For each model, the hazard ratios of BMI at baseline (t = 0 years, represented by the dashed line) and every 5 years afterwards until the maximum lengths of follow-up of the cohort were plotted together. The only exception is for NHIS as the last time point is 8 years instead of 5 or 10 years because the maximum length of follow-up is 9 years.

Very similar changing patterns were shown among four models: women of the Original Cohort of FHS, men of the Original Cohort of FHS, men of NHANES I, and men of NHIS. Plots of all these four models show that the association of low BMI with high mortality is the strongest when the follow-up time is the shortest and it weakens as the follow-up time increases, while the association of high BMI with high mortality is the weakest when the follow-up time is the shortest and it strengthens as the follow-up time increases. The plot for men of the FHS Offspring indicates that the associations of low and high BMI with elevated mortality both strengthen with follow-up time while the plot for women of NHANES II shows the opposite changing trend.

When early deaths within 4 years of follow-up were dropped, most of the estimated Cox models provided results similar to those with early deaths included. The only exception was that no association of BMI and mortality (neither LBMI term was significant) was found for men of the Charleston Heart Study after dropping early deaths. As to the tests of the proportional hazards assumptions, no borderline cases were found and three cohorts maintained significant dynamic associations of BMI and mortality for men: the Original Cohort of FHS, the Offspring Cohort of FHS, and NHANES I. The changing patterns of the hazard functions after dropping early deaths (Additional file 1) are similar to those without dropping early deaths (Figure 1) for these three models for men.

### Impact of length of follow-up on traditional Meta-analysis

**Figure 3:**
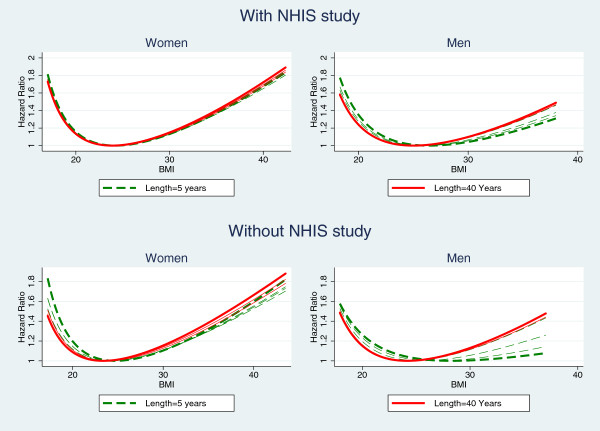
**Hazard ratios of BMI of Cox models based on pooled data with survival times censored at 5, 10, 15, …,40 years.** The hazard ratio at the vertex of each U- or J-shaped curve is 1 (control point). Each line is plotted within the range of 1st - 99th percentiles of baseline BMI of all individuals. The curves for censoring at 5 years (dashed) and 40 years (solid) are in thick lines and those of other lengths are in thin lines. Top two plots are based on data with NHIS cohort and the lower two plots are based on data excluding NHIS that has the largest sample size and the shortest length of follow-up.

The estimated hazard ratios of BMI using Cox models for combined data with censoring at 5, 10, 15, …, 40 years are summarized in Figure 3. The top two plots in Figure 3 show no obvious changes for women and a slight change for men when the length of follow-up increases. Note that NHIS was weighted the most in the combined analyses due to its largest sample size (79.5% of subjects and 52% of deaths in the combined data are from NHIS) yet its maximum survival time is no more than 10 years. The information from NHIS doesn’t change when the survival times are censored at 10 years or longer.

Another analysis excluding NHIS was conducted and the results are in the two plots at the bottom of Figure 3. These plots show more obvious changes in shape as length of follow-up increases. Overall, as the length of follow-up increases, hazard ratio for high BMI increases while that for low BMI decreases and the changes are stronger for men than for women. The association for men is nearly in the inverse direction when the length of follow-up is 5 years. These results are consistent with those based on FHS original cohort in previous studies [25,26].

## Discussions

This study found dynamic associations of BMI and mortality in three cohorts for men and one cohort for women among six different cohorts. This finding is most meaningful from the methodology aspect for epidemiologic research on body weight and mortality.

The traditional analysis models should be used with caution in analyzing the association of BMI and mortality. This study clearly showed that the proportional hazards (PH) assumptions for BMI related terms are not always satisfied. When the PH assumption is violated, that means the underlying instantaneous association of BMI and mortality changes with time and what the Cox model captures will depend on the time period the analysis is based on [26]. A simple analogy is that the average speed of a car (an estimate from the Cox model) will depend on the time period (follow-up period) used for calculating the average speed when the car is driving at a changing speed (dynamic association or non-proportional hazards). When the underlying association is dynamic, results obtained from the same study population using traditional methods such as the Cox models or the logistic regression models are meant to be different when the lengths of follow-up are different. Thus, results from studies of different lengths of follow-up may not be comparable; Meta-analyses based on pooled data may not provide meaningful results without considering the different lengths of follow-up of individual cohorts; large cohorts tend to have shorter lengths of follow-up and may dominate the final results in Meta-analysis.

The health impact of obesity may have been under-estimated. Out of six models showed in Figure 1, three dynamic models for men and one borderline case for women showed similar changing pattern of the association of BMI and mortality with follow-up time: the weakening association of low BMI and the strengthening association of high BMI with mortality. This dynamic feature is consistent with the heterogeneity in the literature using traditional analysis methods. In a large study based on 19 cohorts with different lengths of follow-up, researchers briefly mentioned that “The increased hazard ratios for a BMI below 20.0 as compared with a BMI of 22.5 to 24.9 were reduced as the length of follow-up increased” [32]. In Table 2 of that paper, we can also see that hazard ratios (HRs) for high BMI increased with the length of follow-up. These changing patterns of HRs of low and high BMI are consistent with the changing pattern of the dynamic association identified in this study.

The most controversial finding about BMI and mortality is the inverse association between BMI and mortality. Based on the changing pattern in the four models with similar results in this study, we can see that the inverse association is more likely to be observed in cohorts with short lengths of follow-up. In the literature, findings of inverse associations were all based on relatively short studies (no more than 10 years) [13-17]. All four models for men in Figure 1 showed that the estimated hazard ratios for high BMI increase with time, suggesting that a longer follow-up can allow a study to capture stronger effects of high body weight. Considering that most cohorts used in the literature have relative short lengths of follow-up, especially some large cohorts like NHIS, we might have underestimated the heath impact of obesity, especially for men.

Dropping early deaths doesn’t remove the dynamic association of BMI and mortality. So-called inverse-causality in the literature suggests that individuals with low BMI at baseline may have lost much weight due to aging and/or diseases, or they were smokers [18-21]. Dropping early deaths is a commonly used strategy for handling inverse-causality. In our study, three cohorts supported the dynamic features for men even when deaths in the first 4 years of follow-up were excluded. NHIS is the only study that showed a dynamic association for men before but not after dropping early deaths, but the median length of follow-up for NHIS after dropping early deaths became 3.0 years, which may be too short to capture any significant change. Similar results before and after dropping early deaths indicate that the impact of the dynamic association on findings using traditional analysis methods may not be removed by simply dropping early deaths. This may explain why the previous Meta-analysis on dropping early deaths did not find a significant difference [24].

In in this study, only commonly used covariates (gender, age, smoking status, and race) besides BMI related terms are considered for analysis. Different studies may include other covariates, such as life styles, social economic status, and education etc. These variables are not available in the database used for our study. In the previous mentioned Meta-analysis base on 19 cohorts and 1.46 million subjects, covariates such as alcohol consumption, physical activity, and education were all included for analysis. The evidence of a dynamic association of BMI and mortality can still be seen in the results [32]. It would be very meaningful to add other covariates, especially those related to life style and social economic status, to current model to see if the dynamic feature will be weakened or strengthened, how much of it can be removed by adding certain covariates etc. Such findings may help us understand more about the heterogeneity in the literature since different studies have used different variables to control for the effects of possible confounders. This will be one of the topics for our future research.

There are other limitations for this study. Methodology wise, time-dependent covariates Cox models used in this study are restricted in capturing the nonlinear association and its changes with time. Only a small number of cohorts were considered for analysis: Charleston Heart Study and FHS are relatively small, NHIS is large but its length of follow-up is short. Both small sample sizes and the short length of follow-up limited the power of our study in detecting and modeling dynamic associations. The impact of length of follow-up could not be fully examined by applying censoring times on the pooled data as four cohorts (including 96% of the subjects) had less than 20 years of follow-up.

## Conclusions

Our study found dynamic associations of BMI and mortality in multiple cohorts although not in all six cohorts. The majority of the dynamic associations were found for men and these associations had the same pattern as in the previous study based on FHS [26]. Based on the findings, we conclude that different or even opposite results about body weight and mortality in the literature may be in part due to the underlying dynamic association of BMI and mortality. The dynamic features need to be taken into consideration in future studies.

The importance of identifying the health impact of body weight, the implications of the dynamic associations of BMI and mortality, and the limitations of this study indicate much work is needed for future research. What are the underlying causes of dynamic associations of BMI and mortality? What makes the difference between men and women in terms of the dynamic features? How can we better measure the health impact of obesity with considerations of the dynamic features? These are challenges from both biological and methodological aspects.

## Abbreviations

BMI: Body mass index; NHANES: National Health and Nutrition Examination Survey; FHS: Framingham Heart Study; NHIS: National Health Interview Survey; PH: Proportional hazards; LBMI: Lean BMI =1/BMI.

## Competing interests

The authors declare that they have no competing interests.

## Authors’ contributions

JH proposed the original research idea, designed the study, supervised the data analysis, and wrote the first draft of the manuscript and conducted revisions. QY and HZ participated in the design of the study and conducted data cleaning and analyses. JM contributed significantly to the research idea and design and participated in drafting the manuscript. All authors read and approved the final manuscript.

## Authors’ information

JH is currently an associate professor of the Department of Biostatistics of the University of Kansas Medical Center (KUMC). She got her Ph.D. degree in Statistics from the Florida State University in 2007. Her Ph.D. dissertation is about using Bayesian dynamic survival models for longitudinal data, for which a specific application example is the association of BMI and mortality. JH has extensive statistical and collaborative medical research experience, and has published several manuscripts about innovative analyses of BMI and mortality in epidemiology journals and several manuscripts about relevant innovative methods in statistical journals.

QY is currently a senior research analyst of the Department of Biostatistics of KUMC. She got her Master of Statistics from the University of Missouri Columbia in 2010 and has extensive experience of data analysis for medical and public health research.

HZ is currently a statistical analyst of the Sanofi Biometry Capacity in China. She got her Master degree from the Department of Biostatistics of KUMC in 2013 and has a solid background in statistical analysis and computational statistics. HZ got the Best Student Award (one award in each year) of the Department of Biostatistics of KUMC in 2012 due to her outstanding academic records and excellent work as a graduate research assistant in the Department.

JM is currently an associate professor of the Department of Biostatistics at the University of Kansas Medical Center. He got his Ph.D. in Biometry from the University of Texas-Health Science Center in Huston. JM has extensive statistical methodology research and collaborative medical research experiences, with expertise in study design, secondary data analysis of large databases, and survival analysis etc.

## Supplementary Material

Additional file 1 Model settings and supplementary analyses. Click here for file
