# Supplementary material for: The dynamic association of body mass index and all-cause mortality in multiple cohorts and its impacts
Source: Emerg Themes Epidemiol. 2014 Oct 24;11:17. doi: 10.1186/1742-7622-11-17 (PMC4211318; doi:10.1186/1742-7622-11-17)
Supplement: Additional file 1 — Model settings and supplementary analyses. [file 1742-7622-11-17-S1.doc]

**Additional file 1**

**The Cox model and time-dependent covariate Cox model**

The hazard function of the Cox model is:

$$\begin{matrix} \lambda=\lambda_{0}(t)\exp(Age*\beta_{Age}+Smo\ker*\beta_{smo\ker}+White*\beta_{while}+Hispanic*\beta_{Hispanic} \\ +(LBMI-m)*\beta_{1}+(LBMI-m)^{2}*\beta_{2}), \end{matrix}$$

where$LBMI=1/BMI$and $m$is the mean of LBMI for all individuals considered for each model. LBMI is centered on its mean to reduce the correlation between the linear and quadratic terms of LBMI to avoid the multicollinearity issue.

The scaled hazard function with 1 at the vertex for BMI is:

$\lambda_{BMI}=\exp[\beta_{2}(1/BMI-m+\beta_{1}/(2\beta_{2}))^{2}]$.

The scaled hazard function is indeed the hazard ratio of BMI with the vertex point as the control.

The hazard function of the time-dependent covariate Cox model is:

$$\lambda(t)=\lambda_{0}(t)\exp[(LBMI-m)*(\beta_{1}+t*\beta_{1t})+(LBMI-m)^{2}*(\beta_{2}+t*\beta_{2t})+...],$$

where $t$ is the follow-up time that varies from 0 to the maximum length of follow-up in the cohort.

The scaled hazard function for BMI with 1 at the vertex is:

$\lambda_{BMI}(t)=\exp\{(\beta_{2}+t*\beta_{2t})[1/BMI-m+(\beta_{1}+t*\beta_{1t})/2/(\beta_{2}+t*\beta_{2t})]^{2}\}$.

**Supplementary Analysis I: Dynamic associations of BMI and mortality when deaths within 4 years of follow-up were excluded.**

The same analysis scheme was applied to all 6 cohorts with early deaths in 4 –years dropped. Three models still support dynamic association of BMI and mortality: FHS men, NHANES I men, and FHS Offspring men. The dynamic associations are presented in Figure A2.

Figure A2: Hazard Ratio of BMI for all studies with significant (p<0.05) dynamic associations between BMI and mortality when early deaths within 4 years were excluded. Each line was plotted within the range of 1^st^ - 99^th^ percentiles of BMI of all individuals remain alive at the time point.

**Supplementary Analysis II: Cox model and time-dependent covariates Cox model based on all cohorts pooled together.**

Strong dynamic associations were found for both men and women based on the pooled data.

**Table A1: Cox models and the tests of proportional hazards assumptions**

| Women (death rate=10,920/91,192) | | | | |
| --- | --- | --- | --- | --- |
| Variables | Log hazard ratio  (Standard Error) | Test beta=0  (p-value) | | Test PH assumptions  (p-value) |
| Age (5 years) | 0.463 (0.004) | <0.001 | | <0.001 |
| Smoker (vs. non-smoker) | 0.544 (0.023) | <0.001 | | <0.001 |
| Black (vs. White) | 0.260 (0.028) | <0.001 | | 0.903 |
| Hispanic  (vs. White) | -0.18 (0.13) | 0.175 | | 0.894 |
| LBMIc | -4.628 (1.211) | 0.001 | | <0.001 |
| LBMIc^2^ | 1905.3 (92.8) | <0.001 | | 0.652 |
| Men (death rate=11,205/67,111) | | | | |
| Variables | Log hazard ratio  (Standard Error) | Test beta=0  (p-value) | Test PH assumptions  (p-value) | |
| Age (5 years) | 0.442 (0.004) | <0.001 | <0.001 | |
| Smoker (vs. non-smoker) | 0.539 (0.021) | <0.001 | 0.869 | |
| Black (vs. White) | 0.221 (0.028) | <0.001 | 0.099 | |
| Hispanic  (vs. White) | -0.223 (0.122) | 0.068 | 0.718 | |
| LBMIc | 2.933 (1.545) | 0.058 | <0.001 | |
| LBMIc^2^ | 2131.1 (122.6) | <0.001 | 0.261 | |

LBMIc: lean BMI (1/BMI) centered by its mean of all individuals included in each model.

Cohort effects were estimated but not presented in the table.

**Table A2: Time-dependent covariates Cox models**

| Women (death rate=10,979/92,944) | | |
| --- | --- | --- |
| Variables | Log hazard ratio  (Standard Error) | Test beta=0  (p-value) |
| Age (5 years) | 0.461 (0.004) | <0.001 |
| Smoker (vs. non-smoker) | 0.547 (0.023) | <0.001 |
| Black (vs.White) | 0.264 (0.028) | <0.001 |
| Hispanic (vs. White) | -0.185 (0.131) | 0.158 |
| LBMIc | 6.226 (1.778) | <0.001 |
| LBMIc^2^ | 1802.9 (94.8) | <0.001 |
| LBMIc*t | -0.963 (0.165) | <0.001 |
| Men (death rate=11,273/68,520) | | |
| Variables | Log hazard ratio  (Standard Error) | Test beta=0  (p-value) |
| Age (5 years) | 0.441 (0.004) | <0.001 |
| Smoker (vs. non-smoker) | 0.540 (0.021) | <0.001  0.59 |
| Black (vs.White) | 0.221 (0.028) | <0.001 |
| Hispanic (vs. White) | -0.234 (0.122) | 0.056 |
| LBMIc | 9.712 (2.411) | <0.001 |
| LBMIc^2^ | 2020.4 (124.0) | <0.001 |
| LBMIc*t | -1.47 (0.22) | <0.001 |

LBMIc: lean BMI (1/BMI) centered by its mean of all individuals included in each model.

Cohort effects were also estimated but not presented in the table.

Figure A1. Hazard Ratio of BMI for men and women based on pooled data. Each line was plotted within the range of 1^st^ - 99^th^ percentiles of BMI of all individuals remain alive at the time point.
